# [(4-Methyl­benz­yl)bis­(pyridin-2-ylmeth­yl)amine-κ^3^ *N*,*N*′,*N*′′]bis­(thio­cyanato-κ*S*)copper(II) dichloro­methane hemisolvate

**DOI:** 10.1107/S1600536812011476

**Published:** 2012-03-24

**Authors:** Yan Qi, Yang Li, Zheng-Ping Ma, Qiu-Yun Chen

**Affiliations:** a School of Chemistry and Chemical Engineering, Jiangsu University, Zhenjiang 212013, People’s Republic of China

## Abstract

The title compound, [Cu(NCS)_2_(C_20_H_21_N_3_)]·0.5CH_2_Cl_2_, crystallized with two independent complex mol­ecules (*A* and *B*) in the asymmetric unit, accompanied by one dichloro­methane solvent mol­ecule. Each Cu^II^ atom has a square-pyramidal geometry, being coordinated by five N atoms, three from the (4-methyl­benz­yl)bis­(pyridin-2-ylmeth­yl)amine ligand and two from the thio­cyanate ligands. In the crystal, the *B* mol­ecules are linked *via* C—H⋯S inter­actions, forming chains propagating along [100].

## Related literature

For the synthesis of the (4-methyl-benz­yl)bis­(2-pyridyl-meth­yl)amine ligand, see: Basudeb *et al.* (2009 ▶). For general background to and applications of copper(II) complexes in medicinal chemistry, see: Zhou *et al.* (2011 ▶). For related structures, see: Marti *et al.* (2007 ▶); Chen *et al.* (2008 ▶). For the biological activity of such compounds, see: Chen *et al.* (2011 ▶).
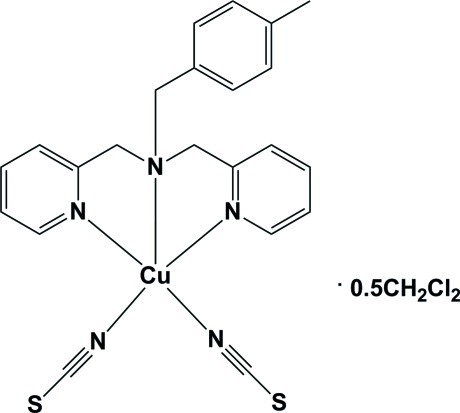


## Experimental

### 

#### Crystal data

[Cu(NCS)_2_(C_20_H_21_N_3_)]·0.5CH_2_Cl_2_
*M* *_r_* = 525.59
Triclinic, 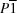

*a* = 10.876 (2) Å
*b* = 12.403 (3) Å
*c* = 19.911 (4) Å
α = 76.13 (3)°
β = 77.01 (3)°
γ = 64.72 (3)°
*V* = 2334.7 (10) Å^3^
*Z* = 4
Mo *K*α radiation
μ = 1.25 mm^−1^
*T* = 293 K
0.20 × 0.20 × 0.20 mm

#### Data collection

Rigaku SCXmini diffractometer
Absorption correction: multi-scan (*CrystalClear*; Rigaku, 2005 ▶) *T*_min_ = 0.955, *T*_max_ = 0.955
20759 measured reflections
8416 independent reflections
6991 reflections with *I* > 2σ(*I*)
*R*_int_ = 0.033

#### Refinement

*R*[*F* ^2^ > 2σ(*F* ^2^)] = 0.055
*wR*(*F* ^2^) = 0.139
*S* = 1.08
8416 reflections
570 parameters
H-atom parameters constrained
Δρ_max_ = 0.75 e Å^−3^
Δρ_min_ = −0.72 e Å^−3^

### 

Data collection: *CrystalClear* (Rigaku, 2005 ▶); cell refinement: *CrystalClear*; data reduction: *CrystalClear*; program(s) used to solve structure: *SHELXS97* (Sheldrick, 2008 ▶); program(s) used to refine structure: *SHELXL97* (Sheldrick, 2008 ▶); molecular graphics: *SHELXTL* (Sheldrick, 2008 ▶); software used to prepare material for publication: *SHELXTL*.

## Supplementary Material

Crystal structure: contains datablock(s) I, global. DOI: 10.1107/S1600536812011476/su2389sup1.cif

Structure factors: contains datablock(s) I. DOI: 10.1107/S1600536812011476/su2389Isup2.hkl

Additional supplementary materials:  crystallographic information; 3D view; checkCIF report

## Figures and Tables

**Table 1:** Hydrogen-bond geometry (Å, °)

*D*—H⋯*A*	*D*—H	H⋯*A*	*D*⋯*A*	*D*—H⋯*A*
C8—H8⋯S2^i^	0.93	2.87	3.503 (5)	127

Symmetry code: (i) 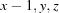.

**Fig. 1.**
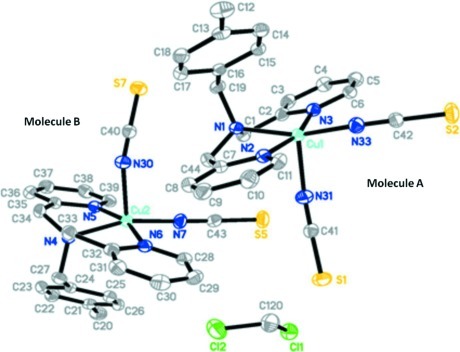
The molecular structure of the two independent molecules (A and B) of the title compound, with the atomic numbering. Displacement ellipsoids are drawn at the 30% probability level [the H atoms have been omitted for clarity].

## supplementary crystallographic information

### Comment

Di(picolyl)amine (dpa) and its derivatives have been used as neutral, non-deprotonated chelating ligands to complex Cu, Zn, Co, and Fe atoms, in order to mimic non-heme dioxygenase or to synthesize metal complexes with open coordination sites (Marti *et al.*, 2007; Chen *et al.*, 2008). A series of metal complexes of N-substituted di(picolyl)amines have been synthesized (Zhou *et al.*, 2011), and some of them have been shown to have anti-cancer activity (Chen *et al.*, 2011). We report herein on the synthesis and crystal structure of the title compound, a copper complex of a dpa type ligand, (4-methyl-benzyl)bis(2-pyridyl-methyl)amine.

The molecular structure of the two indpendent molecules (A and B) of the title compound is shown in Fig. 1. Both copper(II) atoms, Cu1 and Cu2, are coordinated to three N atoms from the organic ligand, and to two N atoms from the thiocyanato ligands. The copper atoms have square pyramidal geometry. The Cu-N distances involving the organic ligand are very similar, varying from 1.998 (3) - 2.039 (4) Å in molecule A, and 2.009 (3) - 2.034 (4)Å in molecule B. The Cu-N(thiocyanato) distances in the equitorial plane are 1.936 (4) and 1.943 (5) Å in molecules A and B, respectively. The Cu-N(thiocyanato) distances in the apical positions are considerably longer, 2.199 (3) and 2.196 (3) Å in molecules A and B, respectively.

In the crystal, the B molecules are linked via a C-H···S interaction (Table 1) forming chains propagating along the a axis direction.

### Experimental

The ligand, (4-methyl-benzyl)bis(2-pyridyl-methyl)amine, was synthesised according to the method described by (Basudeb *et al.*, 2009). The complex was synthesized by adding (4-methyl-benzyl)bis(2-pyridyl-methyl)amine (289 mg, 1 mmol) to a solution of NH_4_SCN (75 mg, 1 mmol) in ethanol (20 ml). The mixture was refluxed at 353 K for 2 h then cooled to room temperature. The solid obtained was filtered off, dried and dissolved in CH_2_Cl_2_, from which colourless block-like crystals were grown by slow evaporation of the solvent at room temperature.

### Refinement

The C-bound H-atoms were included in calculated positions and treated as riding atoms: C-H = 0.93, 0.97 and 0.96 Å for CH, CH_2_ and CH_3_ H-atoms, respectively, with U_iso_(H) = k × U_eq_(parent C-atom), where k = 1.5 for CH_3_ H-atoms and = 1.2 for other H-atoms.

### Crystal data

**Table:**

[Cu(NCS)_2_(C_20_H_21_N_3_)]·0.5CH_2_Cl_2_	*Z* = 4
*M**_r_* = 525.59	*F*(000) = 1080
Triclinic, *P*1	*D*_x_ = 1.495 Mg m^−^^3^
Hall symbol: -P 1	Mo *K*α radiation, λ = 0.71073 Å
*a* = 10.876 (2) Å	Cell parameters from 8552 reflections
*b* = 12.403 (3) Å	θ = 3.3–25.4°
*c* = 19.911 (4) Å	µ = 1.25 mm^−^^1^
α = 76.13 (3)°	*T* = 293 K
β = 77.01 (3)°	Block, colourless
γ = 64.72 (3)°	0.20 × 0.20 × 0.20 mm
*V* = 2334.7 (10) Å^3^	

### Data collection

**Table:**

Rigaku SCXmini diffractometer	8416 independent reflections
Radiation source: fine-focus sealed tube	6991 reflections with *I* > 2σ(*I*)
Graphite monochromator	*R*_int_ = 0.033
CCD_Profile_fitting scans	θ_max_ = 25.4°, θ_min_ = 3.2°
Absorption correction: multi-scan (*CrystalClear*; Rigaku, 2005)	*h* = −12→12
*T*_min_ = 0.955, *T*_max_ = 0.955	*k* = −14→14
20759 measured reflections	*l* = −23→23

### Refinement

**Table:**

Refinement on *F*^2^	Secondary atom site location: difference Fourier map
Least-squares matrix: full	Hydrogen site location: inferred from neighbouring sites
*R*[*F*^2^ > 2σ(*F*^2^)] = 0.055	H-atom parameters constrained
*wR*(*F*^2^) = 0.139	*w* = 1/[σ^2^(*F*_o_^2^) + (0.0587*P*)^2^ + 3.0963*P*] where *P* = (*F*_o_^2^ + 2*F*_c_^2^)/3
*S* = 1.08	(Δ/σ)_max_ < 0.001
8416 reflections	Δρ_max_ = 0.75 e Å^−^^3^
570 parameters	Δρ_min_ = −0.72 e Å^−^^3^
0 restraints	Extinction correction: *SHELXL97* (Sheldrick, 2008)
Primary atom site location: structure-invariant direct methods	Extinction coefficient: 0.473 (17)

### Special details

**Table:**

Geometry. Bond distances, angles etc. have been calculated using the rounded fractional coordinates. All su's are estimated from the variances of the (full) variance-covariance matrix. The cell esds are taken into account in the estimation of distances, angles and torsion angles

### Fractional atomic coordinates and isotropic or equivalent isotropic displacement parameters (Å^2^)

**Table:**

	*x*	*y*	*z*	*U*_iso_*/*U*_eq_	
Cu1	0.62027 (5)	0.89792 (4)	0.31570 (2)	0.0323 (2)	
S1	0.83663 (12)	0.46478 (10)	0.38444 (6)	0.0502 (4)	
S2	0.96179 (13)	0.95101 (13)	0.39115 (8)	0.0620 (5)	
N1	0.4703 (3)	0.9184 (3)	0.26217 (17)	0.0327 (10)	
N2	0.4881 (3)	0.8703 (3)	0.39863 (17)	0.0349 (11)	
N3	0.7056 (3)	0.9454 (3)	0.21854 (17)	0.0347 (10)	
N31	0.7256 (4)	0.7021 (3)	0.3155 (2)	0.0480 (14)	
N33	0.7351 (4)	0.9288 (3)	0.36387 (18)	0.0421 (12)	
C1	0.5457 (4)	0.8759 (4)	0.1955 (2)	0.0387 (12)	
C2	0.6549 (4)	0.9247 (4)	0.1685 (2)	0.0374 (12)	
C3	0.7060 (4)	0.9459 (4)	0.0990 (2)	0.0425 (14)	
C4	0.8089 (4)	0.9897 (4)	0.0797 (2)	0.0444 (14)	
C5	0.8589 (4)	1.0104 (4)	0.1312 (2)	0.0439 (14)	
C6	0.8049 (4)	0.9875 (3)	0.1995 (2)	0.0366 (12)	
C7	0.3924 (4)	0.8427 (3)	0.3824 (2)	0.0366 (14)	
C8	0.2964 (4)	0.8142 (4)	0.4338 (2)	0.0447 (14)	
C9	0.2958 (5)	0.8181 (4)	0.5023 (3)	0.0497 (17)	
C10	0.3918 (5)	0.8490 (4)	0.5185 (2)	0.0479 (17)	
C11	0.4869 (4)	0.8735 (4)	0.4659 (2)	0.0405 (12)	
C12	0.1485 (5)	1.3057 (4)	0.4848 (2)	0.0524 (16)	
C13	0.2085 (4)	1.2361 (3)	0.4243 (2)	0.0392 (11)	
C14	0.3330 (4)	1.2299 (3)	0.3844 (2)	0.0383 (12)	
C15	0.3861 (4)	1.1677 (3)	0.3281 (2)	0.0338 (11)	
C16	0.3150 (4)	1.1118 (3)	0.3094 (2)	0.0320 (11)	
C17	0.1897 (4)	1.1192 (4)	0.3488 (2)	0.0394 (14)	
C18	0.1390 (4)	1.1784 (4)	0.4061 (2)	0.0417 (14)	
C19	0.3708 (4)	1.0475 (3)	0.2471 (2)	0.0375 (12)	
C41	0.7696 (4)	0.6043 (4)	0.3444 (2)	0.0361 (12)	
C42	0.8299 (4)	0.9387 (4)	0.3749 (2)	0.0386 (12)	
C44	0.4000 (4)	0.8402 (4)	0.3066 (2)	0.0417 (14)	
Cu2	0.31226 (5)	0.61598 (4)	0.18480 (3)	0.0332 (2)	
S5	0.77253 (12)	0.56137 (12)	0.13638 (8)	0.0560 (4)	
S7	0.22234 (12)	1.02609 (10)	0.09529 (6)	0.0455 (4)	
N4	0.1335 (3)	0.5956 (3)	0.22734 (17)	0.0337 (10)	
N5	0.2424 (3)	0.6371 (3)	0.09530 (17)	0.0345 (11)	
N6	0.3401 (3)	0.5720 (3)	0.28647 (18)	0.0363 (11)	
N7	0.5035 (4)	0.5832 (3)	0.14723 (19)	0.0433 (12)	
N30	0.2248 (4)	0.8123 (3)	0.1823 (2)	0.0440 (11)	
C20	0.4544 (5)	0.1992 (4)	0.0101 (2)	0.0507 (16)	
C21	0.3765 (4)	0.2710 (3)	0.0684 (2)	0.0384 (14)	
C22	0.2347 (5)	0.3216 (4)	0.0788 (3)	0.0468 (16)	
C23	0.1618 (4)	0.3828 (4)	0.1343 (2)	0.0428 (14)	
C24	0.2284 (4)	0.3978 (3)	0.1809 (2)	0.0343 (11)	
C25	0.3714 (4)	0.3484 (3)	0.1705 (2)	0.0365 (12)	
C26	0.4438 (4)	0.2860 (3)	0.1147 (2)	0.0383 (14)	
C27	0.1507 (4)	0.4645 (3)	0.2411 (2)	0.0387 (12)	
C28	0.4572 (4)	0.5314 (3)	0.3133 (2)	0.0386 (14)	
C29	0.4626 (5)	0.5046 (4)	0.3837 (2)	0.0452 (16)	
C30	0.3410 (5)	0.5214 (4)	0.4292 (2)	0.0482 (16)	
C31	0.2191 (5)	0.5660 (4)	0.4017 (2)	0.0433 (16)	
C32	0.2212 (4)	0.5897 (3)	0.3309 (2)	0.0367 (11)	
C33	0.0948 (4)	0.6391 (4)	0.2951 (2)	0.0390 (12)	
C34	0.0350 (4)	0.6707 (4)	0.1776 (2)	0.0387 (14)	
C35	0.1060 (4)	0.6647 (3)	0.1035 (2)	0.0357 (12)	
C36	0.0389 (5)	0.6867 (4)	0.0470 (2)	0.0432 (14)	
C37	0.1124 (5)	0.6782 (4)	−0.0181 (2)	0.0468 (17)	
C38	0.2528 (5)	0.6468 (4)	−0.0267 (2)	0.0483 (17)	
C39	0.3140 (5)	0.6283 (4)	0.0307 (2)	0.0412 (12)	
C40	0.2236 (4)	0.9003 (4)	0.1454 (2)	0.0361 (12)	
C43	0.6153 (4)	0.5735 (4)	0.1429 (2)	0.0388 (14)	
Cl1	0.97412 (13)	0.18878 (11)	0.20919 (6)	0.0545 (4)	
Cl2	0.68764 (15)	0.25816 (13)	0.27070 (11)	0.0932 (7)	
C120	0.8447 (5)	0.2649 (5)	0.2712 (3)	0.0649 (19)	
H1A	0.48260	0.90320	0.16160	0.0470*	
H1B	0.58700	0.78830	0.20280	0.0470*	
H3	0.67150	0.93090	0.06510	0.0510*	
H4	0.84370	1.00490	0.03290	0.0530*	
H5	0.92830	1.03930	0.11970	0.0530*	
H6	0.83850	1.00190	0.23400	0.0440*	
H8	0.23290	0.79260	0.42210	0.0540*	
H9	0.23130	0.80010	0.53720	0.0590*	
H10	0.39230	0.85320	0.56450	0.0570*	
H11	0.55260	0.89300	0.47720	0.0480*	
H12A	0.22010	1.31580	0.50010	0.0790*	
H12B	0.10740	1.26190	0.52270	0.0790*	
H12C	0.08000	1.38360	0.47020	0.0790*	
H14	0.38150	1.26790	0.39550	0.0460*	
H15	0.47050	1.16340	0.30260	0.0410*	
H17	0.13920	1.08410	0.33650	0.0470*	
H18	0.05670	1.17940	0.43300	0.0500*	
H19A	0.29460	1.04920	0.22870	0.0450*	
H19B	0.41640	1.09150	0.21110	0.0450*	
H44A	0.45010	0.75790	0.29750	0.0500*	
H44B	0.30800	0.86870	0.29500	0.0500*	
H20A	0.45180	0.12040	0.02290	0.0760*	
H20B	0.54800	0.19130	0.00190	0.0760*	
H20C	0.41300	0.24010	−0.03160	0.0760*	
H22	0.18710	0.31460	0.04780	0.0560*	
H23	0.06630	0.41430	0.14040	0.0510*	
H25	0.41900	0.35700	0.20090	0.0440*	
H26	0.53930	0.25390	0.10850	0.0460*	
H27A	0.19830	0.42300	0.28150	0.0470*	
H27B	0.06040	0.46180	0.25250	0.0470*	
H28	0.53850	0.52090	0.28280	0.0460*	
H29	0.54590	0.47580	0.40070	0.0550*	
H30	0.34140	0.50310	0.47730	0.0580*	
H31	0.13630	0.57960	0.43120	0.0520*	
H33A	0.02560	0.61220	0.32420	0.0470*	
H33B	0.05680	0.72680	0.28750	0.0470*	
H34A	−0.00540	0.75390	0.18550	0.0460*	
H34B	−0.03830	0.64250	0.18510	0.0460*	
H36	−0.05530	0.70720	0.05350	0.0520*	
H37	0.06860	0.69330	−0.05650	0.0560*	
H38	0.30470	0.63840	−0.07060	0.0580*	
H39	0.40790	0.60900	0.02480	0.0500*	
H12D	0.83310	0.34880	0.26220	0.0780*	
H12E	0.87250	0.22990	0.31720	0.0780*	

### Atomic displacement parameters (Å^2^)

**Table:**

	*U*^11^	*U*^22^	*U*^33^	*U*^12^	*U*^13^	*U*^23^
Cu1	0.0312 (3)	0.0294 (3)	0.0380 (3)	−0.0113 (2)	−0.0106 (2)	−0.0044 (2)
S1	0.0501 (7)	0.0355 (6)	0.0528 (7)	−0.0108 (5)	−0.0064 (5)	0.0023 (5)
S2	0.0463 (7)	0.0770 (9)	0.0751 (9)	−0.0381 (7)	−0.0205 (6)	0.0028 (7)
N1	0.0335 (18)	0.0259 (16)	0.0399 (19)	−0.0109 (14)	−0.0087 (14)	−0.0060 (13)
N2	0.0349 (18)	0.0298 (17)	0.040 (2)	−0.0114 (14)	−0.0118 (14)	−0.0024 (14)
N3	0.0337 (18)	0.0303 (17)	0.0405 (19)	−0.0093 (14)	−0.0083 (14)	−0.0097 (14)
N31	0.044 (2)	0.033 (2)	0.060 (3)	−0.0090 (17)	−0.0087 (18)	−0.0056 (17)
N33	0.044 (2)	0.045 (2)	0.041 (2)	−0.0206 (17)	−0.0132 (16)	−0.0009 (16)
C1	0.040 (2)	0.038 (2)	0.043 (2)	−0.0131 (19)	−0.0132 (18)	−0.0127 (18)
C2	0.035 (2)	0.033 (2)	0.043 (2)	−0.0063 (17)	−0.0110 (18)	−0.0127 (17)
C3	0.042 (2)	0.043 (2)	0.041 (3)	−0.007 (2)	−0.0153 (19)	−0.0141 (19)
C4	0.043 (3)	0.047 (2)	0.036 (2)	−0.013 (2)	−0.0001 (18)	−0.0082 (19)
C5	0.040 (2)	0.043 (2)	0.048 (3)	−0.016 (2)	−0.004 (2)	−0.009 (2)
C6	0.036 (2)	0.033 (2)	0.043 (2)	−0.0125 (18)	−0.0097 (18)	−0.0082 (17)
C7	0.034 (2)	0.0259 (19)	0.047 (3)	−0.0101 (17)	−0.0099 (18)	−0.0001 (17)
C8	0.037 (2)	0.037 (2)	0.060 (3)	−0.0160 (19)	−0.011 (2)	−0.001 (2)
C9	0.038 (3)	0.045 (3)	0.054 (3)	−0.012 (2)	−0.003 (2)	0.002 (2)
C10	0.045 (3)	0.054 (3)	0.041 (3)	−0.016 (2)	−0.008 (2)	−0.006 (2)
C11	0.035 (2)	0.042 (2)	0.041 (2)	−0.0095 (19)	−0.0125 (18)	−0.0048 (18)
C12	0.059 (3)	0.039 (2)	0.048 (3)	−0.011 (2)	−0.003 (2)	−0.007 (2)
C13	0.044 (2)	0.0269 (19)	0.038 (2)	−0.0070 (18)	−0.0107 (18)	0.0017 (16)
C14	0.040 (2)	0.031 (2)	0.044 (2)	−0.0111 (18)	−0.0117 (18)	−0.0065 (17)
C15	0.031 (2)	0.0302 (19)	0.037 (2)	−0.0104 (17)	−0.0070 (16)	−0.0007 (16)
C16	0.032 (2)	0.0260 (18)	0.036 (2)	−0.0077 (16)	−0.0130 (16)	−0.0008 (15)
C17	0.033 (2)	0.035 (2)	0.050 (3)	−0.0124 (18)	−0.0105 (18)	−0.0042 (18)
C18	0.031 (2)	0.037 (2)	0.051 (3)	−0.0120 (18)	−0.0006 (18)	−0.0037 (19)
C19	0.037 (2)	0.032 (2)	0.040 (2)	−0.0081 (18)	−0.0122 (18)	−0.0040 (17)
C41	0.031 (2)	0.036 (2)	0.039 (2)	−0.0091 (18)	−0.0025 (17)	−0.0119 (18)
C42	0.042 (2)	0.040 (2)	0.037 (2)	−0.021 (2)	−0.0121 (18)	0.0034 (18)
C44	0.039 (2)	0.037 (2)	0.055 (3)	−0.0187 (19)	−0.013 (2)	−0.0050 (19)
Cu2	0.0267 (3)	0.0301 (3)	0.0395 (3)	−0.0111 (2)	−0.0011 (2)	−0.0033 (2)
S5	0.0311 (6)	0.0610 (8)	0.0791 (9)	−0.0172 (6)	−0.0084 (6)	−0.0182 (7)
S7	0.0531 (7)	0.0420 (6)	0.0411 (6)	−0.0220 (5)	−0.0072 (5)	0.0003 (5)
N4	0.0284 (17)	0.0307 (17)	0.0375 (19)	−0.0075 (14)	−0.0028 (14)	−0.0070 (14)
N5	0.0343 (19)	0.0279 (16)	0.040 (2)	−0.0139 (14)	−0.0033 (14)	−0.0018 (14)
N6	0.0327 (19)	0.0281 (16)	0.044 (2)	−0.0109 (14)	−0.0014 (15)	−0.0046 (14)
N7	0.028 (2)	0.049 (2)	0.046 (2)	−0.0127 (16)	−0.0023 (15)	−0.0027 (17)
N30	0.046 (2)	0.0334 (19)	0.054 (2)	−0.0161 (17)	−0.0113 (17)	−0.0048 (17)
C20	0.061 (3)	0.039 (2)	0.047 (3)	−0.016 (2)	−0.001 (2)	−0.011 (2)
C21	0.045 (3)	0.0268 (19)	0.039 (2)	−0.0123 (18)	−0.0038 (18)	−0.0030 (17)
C22	0.046 (3)	0.037 (2)	0.059 (3)	−0.013 (2)	−0.013 (2)	−0.012 (2)
C23	0.033 (2)	0.034 (2)	0.062 (3)	−0.0146 (18)	−0.005 (2)	−0.008 (2)
C24	0.033 (2)	0.0270 (19)	0.040 (2)	−0.0138 (17)	−0.0042 (17)	0.0024 (16)
C25	0.038 (2)	0.032 (2)	0.041 (2)	−0.0159 (18)	−0.0076 (18)	−0.0027 (17)
C26	0.029 (2)	0.032 (2)	0.049 (3)	−0.0112 (17)	−0.0011 (18)	−0.0033 (18)
C27	0.040 (2)	0.030 (2)	0.045 (2)	−0.0167 (18)	0.0015 (18)	−0.0057 (17)
C28	0.039 (2)	0.028 (2)	0.046 (3)	−0.0110 (18)	−0.0058 (18)	−0.0055 (17)
C29	0.048 (3)	0.038 (2)	0.048 (3)	−0.012 (2)	−0.016 (2)	−0.0049 (19)
C30	0.065 (3)	0.044 (2)	0.038 (3)	−0.023 (2)	−0.004 (2)	−0.011 (2)
C31	0.043 (3)	0.041 (2)	0.046 (3)	−0.017 (2)	0.005 (2)	−0.0170 (19)
C32	0.039 (2)	0.0279 (19)	0.039 (2)	−0.0101 (17)	0.0004 (18)	−0.0092 (17)
C33	0.032 (2)	0.038 (2)	0.045 (2)	−0.0130 (18)	0.0020 (18)	−0.0117 (18)
C34	0.026 (2)	0.038 (2)	0.050 (3)	−0.0103 (17)	−0.0032 (17)	−0.0099 (18)
C35	0.034 (2)	0.030 (2)	0.044 (2)	−0.0149 (17)	−0.0052 (17)	−0.0037 (17)
C36	0.039 (2)	0.039 (2)	0.050 (3)	−0.0131 (19)	−0.012 (2)	−0.0033 (19)
C37	0.054 (3)	0.047 (3)	0.041 (3)	−0.022 (2)	−0.011 (2)	−0.002 (2)
C38	0.056 (3)	0.050 (3)	0.042 (3)	−0.029 (2)	−0.001 (2)	−0.003 (2)
C39	0.044 (2)	0.038 (2)	0.041 (2)	−0.020 (2)	−0.0002 (19)	−0.0032 (18)
C40	0.036 (2)	0.037 (2)	0.039 (2)	−0.0132 (18)	−0.0062 (17)	−0.0143 (18)
C43	0.038 (3)	0.034 (2)	0.037 (2)	−0.0088 (18)	−0.0038 (18)	−0.0045 (17)
Cl1	0.0532 (7)	0.0594 (7)	0.0503 (7)	−0.0226 (6)	−0.0035 (5)	−0.0104 (5)
Cl2	0.0510 (8)	0.0574 (9)	0.1673 (18)	−0.0196 (7)	0.0141 (9)	−0.0420 (10)
C120	0.055 (3)	0.068 (3)	0.066 (4)	−0.019 (3)	−0.001 (3)	−0.018 (3)

### Geometric parameters (Å, º)

**Table:**

Cu1—N1	2.039 (4)	C6—H6	0.9300
Cu1—N2	1.998 (3)	C8—H8	0.9300
Cu1—N3	2.016 (3)	C9—H9	0.9300
Cu1—N31	2.199 (3)	C10—H10	0.9300
Cu1—N33	1.936 (4)	C11—H11	0.9300
Cu2—N7	1.943 (5)	C12—H12A	0.9600
Cu2—N30	2.196 (3)	C12—H12B	0.9600
Cu2—N5	2.009 (3)	C12—H12C	0.9600
Cu2—N6	2.023 (4)	C14—H14	0.9300
Cu2—N4	2.034 (4)	C15—H15	0.9300
Cl1—C120	1.744 (6)	C17—H17	0.9300
Cl2—C120	1.748 (6)	C18—H18	0.9300
S1—C41	1.638 (5)	C19—H19B	0.9700
S2—C42	1.614 (5)	C19—H19A	0.9700
S5—C43	1.629 (5)	C44—H44A	0.9700
S7—C40	1.634 (4)	C44—H44B	0.9700
N1—C19	1.504 (5)	C20—C21	1.506 (6)
N1—C1	1.480 (5)	C21—C26	1.386 (6)
N1—C44	1.492 (6)	C21—C22	1.381 (7)
N2—C11	1.347 (5)	C22—C23	1.386 (7)
N2—C7	1.347 (6)	C23—C24	1.386 (6)
N3—C2	1.361 (6)	C24—C25	1.393 (6)
N3—C6	1.336 (6)	C24—C27	1.503 (5)
N31—C41	1.151 (6)	C25—C26	1.397 (5)
N33—C42	1.162 (7)	C28—C29	1.370 (5)
N4—C34	1.476 (6)	C29—C30	1.389 (7)
N4—C33	1.479 (5)	C30—C31	1.385 (8)
N4—C27	1.518 (5)	C31—C32	1.366 (5)
N5—C39	1.348 (5)	C32—C33	1.512 (6)
N5—C35	1.352 (6)	C34—C35	1.507 (6)
N6—C28	1.338 (6)	C35—C36	1.387 (6)
N6—C32	1.356 (6)	C36—C37	1.363 (6)
N7—C43	1.155 (7)	C37—C38	1.385 (8)
N30—C40	1.157 (6)	C38—C39	1.374 (7)
C1—C2	1.496 (7)	C20—H20B	0.9600
C2—C3	1.378 (6)	C20—H20C	0.9600
C3—C4	1.386 (7)	C20—H20A	0.9600
C4—C5	1.382 (6)	C22—H22	0.9300
C5—C6	1.369 (6)	C23—H23	0.9300
C7—C8	1.387 (6)	C25—H25	0.9300
C7—C44	1.500 (5)	C26—H26	0.9300
C8—C9	1.375 (7)	C27—H27A	0.9700
C9—C10	1.376 (8)	C27—H27B	0.9700
C10—C11	1.374 (7)	C28—H28	0.9300
C12—C13	1.512 (6)	C29—H29	0.9300
C13—C14	1.388 (6)	C30—H30	0.9300
C13—C18	1.387 (6)	C31—H31	0.9300
C14—C15	1.389 (5)	C33—H33A	0.9700
C15—C16	1.389 (6)	C33—H33B	0.9700
C16—C19	1.505 (5)	C34—H34B	0.9700
C16—C17	1.389 (6)	C34—H34A	0.9700
C17—C18	1.385 (6)	C36—H36	0.9300
C1—H1B	0.9700	C37—H37	0.9300
C1—H1A	0.9700	C38—H38	0.9300
C3—H3	0.9300	C39—H39	0.9300
C4—H4	0.9300	C120—H12D	0.9700
C5—H5	0.9300	C120—H12E	0.9700
			
N1—Cu1—N2	82.85 (14)	H12A—C12—H12C	109.00
N1—Cu1—N3	81.51 (14)	H12B—C12—H12C	109.00
N1—Cu1—N31	94.06 (16)	C13—C12—H12A	109.00
N1—Cu1—N33	163.37 (15)	C13—C14—H14	120.00
N2—Cu1—N3	163.69 (15)	C15—C14—H14	120.00
N2—Cu1—N31	90.42 (15)	C14—C15—H15	119.00
N2—Cu1—N33	97.94 (16)	C16—C15—H15	120.00
N3—Cu1—N31	95.27 (15)	C16—C17—H17	120.00
N3—Cu1—N33	95.74 (16)	C18—C17—H17	120.00
N31—Cu1—N33	102.53 (16)	C13—C18—H18	119.00
N5—Cu2—N30	91.31 (15)	C17—C18—H18	119.00
N6—Cu2—N7	95.90 (16)	N1—C19—H19A	109.00
N6—Cu2—N30	95.54 (15)	C16—C19—H19B	109.00
N7—Cu2—N30	102.16 (16)	H19A—C19—H19B	108.00
N4—Cu2—N6	81.28 (15)	C16—C19—H19A	109.00
N4—Cu2—N7	162.81 (15)	N1—C19—H19B	109.00
N4—Cu2—N30	95.00 (16)	N1—C44—H44B	110.00
N5—Cu2—N6	162.51 (15)	N1—C44—H44A	110.00
N5—Cu2—N7	98.37 (16)	H44A—C44—H44B	108.00
N4—Cu2—N5	82.11 (14)	C7—C44—H44A	110.00
Cu1—N1—C1	104.3 (3)	C7—C44—H44B	110.00
C1—N1—C44	113.2 (3)	C20—C21—C22	120.9 (4)
C19—N1—C44	111.4 (3)	C20—C21—C26	121.5 (4)
C1—N1—C19	109.2 (3)	C22—C21—C26	117.5 (4)
Cu1—N1—C19	113.1 (3)	C21—C22—C23	121.5 (5)
Cu1—N1—C44	105.5 (2)	C22—C23—C24	121.3 (5)
Cu1—N2—C7	113.0 (3)	C23—C24—C25	117.8 (4)
Cu1—N2—C11	128.2 (3)	C23—C24—C27	121.9 (4)
C7—N2—C11	118.8 (4)	C25—C24—C27	120.4 (4)
Cu1—N3—C2	112.1 (3)	C24—C25—C26	120.4 (4)
C2—N3—C6	119.3 (3)	C21—C26—C25	121.5 (4)
Cu1—N3—C6	128.5 (3)	N4—C27—C24	114.2 (3)
Cu1—N31—C41	151.1 (3)	N6—C28—C29	122.9 (4)
Cu1—N33—C42	159.9 (3)	C28—C29—C30	118.5 (5)
C27—N4—C34	111.7 (3)	C29—C30—C31	118.8 (4)
Cu2—N4—C34	106.0 (3)	C30—C31—C32	119.6 (5)
C27—N4—C33	108.5 (3)	N6—C32—C31	121.6 (4)
Cu2—N4—C27	112.4 (3)	N6—C32—C33	114.2 (3)
Cu2—N4—C33	105.0 (3)	C31—C32—C33	124.2 (4)
C33—N4—C34	113.1 (3)	N4—C33—C32	108.9 (4)
C35—N5—C39	118.6 (4)	N4—C34—C35	110.5 (4)
Cu2—N5—C35	113.4 (3)	N5—C35—C34	114.8 (4)
Cu2—N5—C39	128.0 (3)	N5—C35—C36	121.5 (4)
C28—N6—C32	118.6 (4)	C34—C35—C36	123.7 (4)
Cu2—N6—C28	128.4 (3)	C35—C36—C37	119.3 (5)
Cu2—N6—C32	113.0 (3)	C36—C37—C38	119.6 (4)
Cu2—N7—C43	160.1 (3)	C37—C38—C39	118.9 (4)
Cu2—N30—C40	140.7 (3)	N5—C39—C38	122.2 (5)
N1—C1—C2	109.4 (4)	S7—C40—N30	178.3 (4)
N3—C2—C1	114.9 (3)	S5—C43—N7	179.4 (4)
C1—C2—C3	124.6 (4)	C21—C20—H20A	109.00
N3—C2—C3	120.5 (4)	C21—C20—H20B	109.00
C2—C3—C4	119.9 (4)	C21—C20—H20C	109.00
C3—C4—C5	118.8 (4)	H20A—C20—H20B	109.00
C4—C5—C6	119.0 (4)	H20A—C20—H20C	110.00
N3—C6—C5	122.6 (4)	H20B—C20—H20C	109.00
C8—C7—C44	122.7 (4)	C21—C22—H22	119.00
N2—C7—C8	121.2 (4)	C23—C22—H22	119.00
N2—C7—C44	116.1 (4)	C22—C23—H23	119.00
C7—C8—C9	119.5 (5)	C24—C23—H23	119.00
C8—C9—C10	119.1 (5)	C24—C25—H25	120.00
C9—C10—C11	119.1 (4)	C26—C25—H25	120.00
N2—C11—C10	122.2 (4)	C21—C26—H26	119.00
C14—C13—C18	117.9 (4)	C25—C26—H26	119.00
C12—C13—C14	121.3 (4)	N4—C27—H27A	109.00
C12—C13—C18	120.8 (4)	N4—C27—H27B	109.00
C13—C14—C15	120.9 (4)	C24—C27—H27A	109.00
C14—C15—C16	120.9 (4)	C24—C27—H27B	109.00
C15—C16—C17	118.2 (4)	H27A—C27—H27B	108.00
C15—C16—C19	120.8 (4)	N6—C28—H28	119.00
C17—C16—C19	121.0 (4)	C29—C28—H28	119.00
C16—C17—C18	120.7 (4)	C28—C29—H29	121.00
C13—C18—C17	121.3 (4)	C30—C29—H29	121.00
N1—C19—C16	114.4 (3)	C29—C30—H30	121.00
S1—C41—N31	178.4 (5)	C31—C30—H30	121.00
S2—C42—N33	179.2 (4)	C30—C31—H31	120.00
N1—C44—C7	110.2 (4)	C32—C31—H31	120.00
H1A—C1—H1B	108.00	N4—C33—H33A	110.00
N1—C1—H1A	110.00	N4—C33—H33B	110.00
N1—C1—H1B	110.00	C32—C33—H33A	110.00
C2—C1—H1A	110.00	C32—C33—H33B	110.00
C2—C1—H1B	110.00	H33A—C33—H33B	108.00
C2—C3—H3	120.00	N4—C34—H34A	110.00
C4—C3—H3	120.00	N4—C34—H34B	110.00
C3—C4—H4	121.00	C35—C34—H34A	110.00
C5—C4—H4	121.00	C35—C34—H34B	110.00
C6—C5—H5	120.00	H34A—C34—H34B	108.00
C4—C5—H5	121.00	C35—C36—H36	120.00
N3—C6—H6	119.00	C37—C36—H36	120.00
C5—C6—H6	119.00	C36—C37—H37	120.00
C7—C8—H8	120.00	C38—C37—H37	120.00
C9—C8—H8	120.00	C37—C38—H38	121.00
C8—C9—H9	120.00	C39—C38—H38	121.00
C10—C9—H9	120.00	N5—C39—H39	119.00
C9—C10—H10	120.00	C38—C39—H39	119.00
C11—C10—H10	120.00	Cl1—C120—Cl2	112.7 (3)
N2—C11—H11	119.00	Cl1—C120—H12D	109.00
C10—C11—H11	119.00	Cl1—C120—H12E	109.00
C13—C12—H12B	110.00	Cl2—C120—H12D	109.00
C13—C12—H12C	110.00	Cl2—C120—H12E	109.00
H12A—C12—H12B	109.00	H12D—C120—H12E	108.00
			
N2—Cu1—N1—C1	−149.3 (3)	C27—N4—C34—C35	−85.1 (4)
N3—Cu1—N1—C1	35.4 (3)	Cu2—N4—C34—C35	37.7 (4)
N31—Cu1—N1—C1	−59.3 (3)	C34—N4—C33—C32	−158.9 (3)
N2—Cu1—N1—C19	92.2 (3)	Cu2—N4—C27—C24	−47.4 (4)
N3—Cu1—N1—C19	−83.1 (3)	C33—N4—C27—C24	−163.1 (4)
N31—Cu1—N1—C19	−177.9 (3)	C33—N4—C34—C35	152.3 (4)
N2—Cu1—N1—C44	−29.8 (3)	C27—N4—C33—C32	76.6 (4)
N3—Cu1—N1—C44	154.9 (3)	C34—N4—C27—C24	71.6 (5)
N31—Cu1—N1—C44	60.1 (3)	Cu2—N4—C33—C32	−43.8 (4)
N1—Cu1—N2—C7	18.2 (3)	Cu2—N5—C35—C36	177.7 (3)
N31—Cu1—N2—C7	−75.8 (3)	C39—N5—C35—C34	179.1 (4)
N33—Cu1—N2—C7	−178.6 (3)	Cu2—N5—C39—C38	−179.0 (3)
N1—Cu1—N2—C11	−163.4 (4)	C39—N5—C35—C36	−1.6 (6)
N31—Cu1—N2—C11	102.5 (4)	C35—N5—C39—C38	0.2 (6)
N33—Cu1—N2—C11	−0.2 (4)	Cu2—N5—C35—C34	−1.6 (4)
N1—Cu1—N3—C2	−21.7 (3)	C28—N6—C32—C33	178.3 (3)
N31—Cu1—N3—C2	71.6 (3)	Cu2—N6—C32—C33	−0.6 (4)
N33—Cu1—N3—C2	174.8 (3)	C32—N6—C28—C29	1.4 (6)
N1—Cu1—N3—C6	161.4 (4)	C28—N6—C32—C31	−0.6 (6)
N31—Cu1—N3—C6	−105.3 (4)	Cu2—N6—C32—C31	−179.5 (3)
N33—Cu1—N3—C6	−2.1 (4)	Cu2—N6—C28—C29	−180.0 (3)
N1—Cu1—N31—C41	−124.4 (9)	N1—C1—C2—N3	28.8 (5)
N2—Cu1—N31—C41	−41.6 (9)	N1—C1—C2—C3	−152.7 (4)
N3—Cu1—N31—C41	153.7 (9)	N3—C2—C3—C4	−0.6 (7)
N33—Cu1—N31—C41	56.6 (9)	C1—C2—C3—C4	−179.0 (4)
N2—Cu1—N33—C42	160.3 (10)	C2—C3—C4—C5	0.5 (7)
N3—Cu1—N33—C42	−28.7 (10)	C3—C4—C5—C6	−0.3 (7)
N31—Cu1—N33—C42	68.0 (10)	C4—C5—C6—N3	0.2 (6)
N7—Cu2—N6—C28	−1.5 (4)	N2—C7—C8—C9	2.2 (6)
N30—Cu2—N6—C28	−104.4 (4)	C8—C7—C44—N1	157.7 (4)
N4—Cu2—N6—C32	−19.9 (3)	N2—C7—C44—N1	−24.6 (5)
N7—Cu2—N6—C32	177.2 (3)	C44—C7—C8—C9	179.8 (4)
N30—Cu2—N6—C32	74.3 (3)	C7—C8—C9—C10	−0.7 (7)
N4—Cu2—N30—C40	−134.1 (6)	C8—C9—C10—C11	−0.9 (7)
N5—Cu2—N30—C40	−51.9 (6)	C9—C10—C11—N2	1.1 (7)
N6—Cu2—N30—C40	144.2 (6)	C18—C13—C14—C15	−0.1 (6)
N7—Cu2—N30—C40	46.9 (7)	C12—C13—C14—C15	−178.8 (4)
N30—Cu2—N4—C34	59.9 (3)	C14—C13—C18—C17	−1.8 (6)
N4—Cu2—N5—C35	18.8 (3)	C12—C13—C18—C17	176.9 (4)
N7—Cu2—N5—C35	−178.6 (3)	C13—C14—C15—C16	1.2 (6)
N30—Cu2—N5—C35	−76.1 (3)	C14—C15—C16—C17	−0.4 (6)
N4—Cu2—N5—C39	−162.0 (4)	C14—C15—C16—C19	178.2 (3)
N7—Cu2—N5—C39	0.7 (4)	C15—C16—C17—C18	−1.5 (6)
N30—Cu2—N5—C39	103.1 (4)	C15—C16—C19—N1	85.7 (5)
N5—Cu2—N4—C27	91.6 (3)	C19—C16—C17—C18	179.9 (4)
N6—Cu2—N4—C27	−82.9 (3)	C17—C16—C19—N1	−95.8 (5)
N30—Cu2—N4—C27	−177.8 (3)	C16—C17—C18—C13	2.6 (7)
N5—Cu2—N4—C33	−150.7 (3)	C20—C21—C26—C25	−177.3 (4)
N6—Cu2—N4—C33	34.8 (3)	C26—C21—C22—C23	−1.5 (7)
N30—Cu2—N4—C33	−60.0 (3)	C20—C21—C22—C23	176.8 (4)
N5—Cu2—N4—C34	−30.7 (3)	C22—C21—C26—C25	0.9 (6)
N6—Cu2—N4—C34	154.8 (3)	C21—C22—C23—C24	1.3 (7)
N4—Cu2—N6—C28	161.4 (4)	C22—C23—C24—C25	−0.5 (6)
Cu1—N1—C19—C16	−49.0 (4)	C22—C23—C24—C27	179.7 (4)
C1—N1—C19—C16	−164.6 (4)	C27—C24—C25—C26	179.8 (3)
C44—N1—C19—C16	69.5 (5)	C23—C24—C27—N4	−97.4 (5)
Cu1—N1—C44—C7	36.4 (4)	C23—C24—C25—C26	0.0 (6)
C1—N1—C44—C7	149.8 (4)	C25—C24—C27—N4	82.8 (4)
C19—N1—C44—C7	−86.6 (4)	C24—C25—C26—C21	−0.2 (6)
C44—N1—C1—C2	−157.4 (4)	N6—C28—C29—C30	−0.6 (6)
Cu1—N1—C1—C2	−43.4 (4)	C28—C29—C30—C31	−0.9 (7)
C19—N1—C1—C2	77.8 (4)	C29—C30—C31—C32	1.5 (7)
C7—N2—C11—C10	0.3 (6)	C30—C31—C32—N6	−0.8 (6)
C11—N2—C7—C44	−179.7 (4)	C30—C31—C32—C33	−179.6 (4)
Cu1—N2—C11—C10	−178.0 (3)	C31—C32—C33—N4	−150.8 (4)
Cu1—N2—C7—C8	176.5 (3)	N6—C32—C33—N4	30.4 (5)
C11—N2—C7—C8	−2.0 (6)	N4—C34—C35—C36	155.8 (4)
Cu1—N2—C7—C44	−1.2 (4)	N4—C34—C35—N5	−25.0 (5)
C6—N3—C2—C3	0.5 (6)	C34—C35—C36—C37	−179.5 (4)
C6—N3—C2—C1	179.1 (4)	N5—C35—C36—C37	1.2 (6)
Cu1—N3—C2—C3	−176.7 (3)	C35—C36—C37—C38	0.5 (7)
C2—N3—C6—C5	−0.3 (6)	C36—C37—C38—C39	−1.8 (7)
Cu1—N3—C2—C1	1.9 (5)	C37—C38—C39—N5	1.5 (7)
Cu1—N3—C6—C5	176.4 (3)		

### Hydrogen-bond geometry (Å, º)

**Table:**

*D*—H···*A*	*D*—H	H···*A*	*D*···*A*	*D*—H···*A*
C8—H8···S2^i^	0.93	2.87	3.503 (5)	127

Symmetry code: (i) *x*−1, *y*, *z*.
